# Pneumopathie grave avec atteinte bronchique compliquant une varicelle chez un adulte immunocompétent

**DOI:** 10.11604/pamj.2014.19.208.5246

**Published:** 2014-10-27

**Authors:** Issam Serghini, Khalid Chkoura, Nawfal Hjira, Mohamed Zoubir, Lalaoui, Mohamed Boughalem

**Affiliations:** 1 Pôle Anesthésie-Réanimation, Hôpital Militaire Avicenne, Faculté de Médecine et de Pharmacie, Université Cadi Ayyad, 40010 Marrakech, Maroc; 2 Service de Dermatologie, 5^e^ Hôpital Militaire Guelmim, Maroc

**Keywords:** Varicelle, pneumonie virale, endoscopie bronchique, Aciclovir, varicella, viral pneumonia, bronchoscopy, Aciclovir

## Abstract

La varicelle est une infection virale cosmopolite, très contagieuse, due au virus varicelle-zona (VZV) et caractérisée par de la fièvre et une éruption papulo-vésiculeuse prurigineuse. L'incidence de la varicelle a significativement augmenté dans les dernières décennies en Europe et aux États-Unis. Chez l'enfant, la varicelle est une infection habituellement bénigne. Chez l'adulte, son évolution peut être émaillée de complications. La pneumonie varicelleuse est la plus fréquente des complications graves de la varicelle chez l'adulte, avec une incidence estimée de 16 à 33% et une mortalité pouvant atteindre 20%. Nous rapportons un cas de varicelle compliquée d'une pneumopathie hypoxémiante. L'examen endoscopique bronchique met en évidence des lésions vésiculeuses de la muqueuse bronchique. Sous traitement antiviral, l’évolution est favorable.

## Introduction

La varicelle est une maladie éruptive très fréquente et habituellement bénigne chez l'enfant sain. Chez l'adulte, elle peut s'accompagner de complications multiviscérales, dont la pneumonie varicelleuse est la plus fréquente. L'incidence de l'atteinte pulmonaire est estimée entre 5 et 50% [1]. Le risque d'atteinte pulmonaire au cours de la varicelle est 25 fois plus élevé chez l'adulte que chez l'enfant [1]. Nous rapportons le cas d'une Pneumopathie grave avec atteinte bronchique compliquant une varicelle chez un adulte immunocompétent

## Patient et observation

Il s'agit d'un patient de 43 ans, tabagique à 18 PA, sans terrain d'immunodépression sous-jacent connu, sans antécédent de varicelle dans l'enfance, ayant une fille de huit ans traité pour varicelle cutanée depuis quinze jours. L'histoire de sa maladie débutait neuf jours avant son hospitalisation, par l'installation brutale d'un syndrome pseudogrippal: myalgies, arthralgies et fiévre. Le lendemain apparaissait une éruption érythémato-vésiculeuse prurigineuse débutant au niveau du thorax et la face puis s’étendant à l'ensemble du revêtement cutané. Le diagnostic d'une varicelle était porté en ville. L’évolution était marquée par l'apparition, deux jours plus tard d'une dyspnée d'aggravation rapide, accompagnée d'une toux ramenant des expectorations purulentes parfois hémoptoïques, avec une altération de l’état général. Il consultait les urgences du 5 ^e^ hopital militaire de Guelmim.

**Figure 1:**
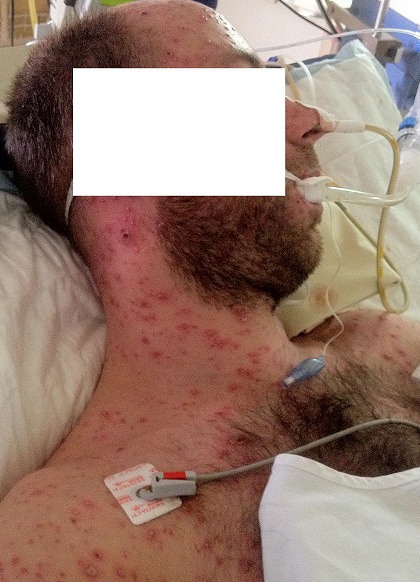
Éruption érythémato-vésiculeuse prurigineuse au niveau du thorax et la face

**Figure 2:**
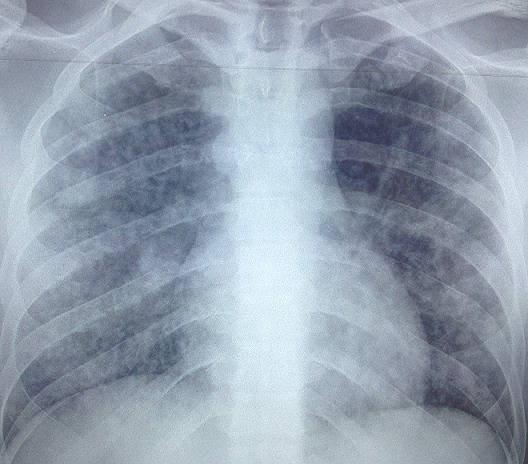
Radiographie du thorax de face qui montre la présence d'opacités alvéolo-interstitielles bilatérales et diffuses aux deux champs pulmonaires

**Figure 3:**
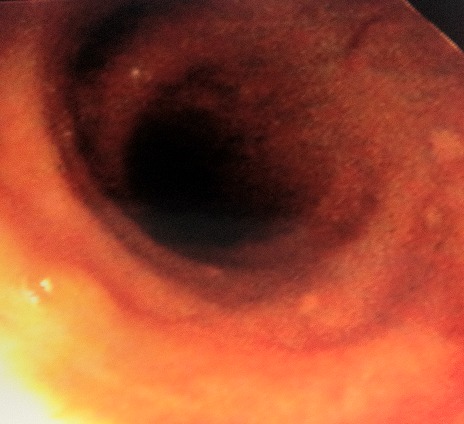
Aspect endobronchique montrant une vésicule sur la bronche intermédiaire

L'examen clinique à l'admission trouvait une température de 39,2°C, une pression artérielle à 110/70 mmHg, une polypnée à 34 cycles/min, une cyanose des extrémités, des signes de lutte respiratoire, une saturation percutanée en oxygène à 77% en air ambiant, une fréquence cardiaque à 120 battements par minute sans signes d'insuffisance circulatoire aiguë et à l'auscultation pulmonaire, des râles crépitants diffus aux deux champs. L’éruption cutanée était faite de lésions diffuses à tout le revêtement cutané faites de vésicules qui se desséchaient et devenaient croûteuses (Figure 1). Le reste de l'examen somatique était sans particularités. Les données biologiques montraient à la gazométrie artérielle une hypoxémie sévère (PaO2 = 43 mmHg, pH = 7, 32, SaO2 = 82%). Il était transféré en réanimation, intubé et mis sous ventilation mécanique en mode ventilation assistée contrôlée (VAC) (fraction en oxygène des gaz inspirés (FiO2) à 100%, pression expiratoire positive (PEEP) à +6). l'aspiration sur tube était très productive et hémorragique. Le reste des examens paracliniques retrouvait un bilan hydroélectrolytique et une fonction rénale normale, une cytolyse hépatique modérée (ALAT 140 UI/l). La CRP était à 88 mg/L. La formule sanguine montrait une thrombopénie à 91 000/mm^3^ et une leucocytose à 19600/mm^3^. La sérologie VIH était négative et la sérologie pour le varicelle-zona-virus (VZV) était positive à IgM. La radiographie thoracique (Figure 2) révélait de multiples opacités micronodulaires et nodulaires bilatérales, diffuses à l'ensemble du parenchyme pulmonaire et confluentes par endroits. La fibroscopie bronchique a mis en évidence dans les bronches lobaires une dizaine de vésicules endobronchiques d'environ 3 mm de diamètre, à contenu clair pour certaines, trouble pour d'autres, et des lésions d’érosion muqueuse d'aspect cicatriciel (Figure 3). Les biopsies bronchiques de ces vésicules ou leurs marges ne sont pas réalisées. Les recherches bactériologiques et myco-parasitologiques sur le produit d'aspiration bronchique et le lavage broncho-alvéolaire sont négatives. Il n'a pas été possible de réaliser de PCR ou de culture spécifique pour le VZV sur ces prélèvements. Le diagnostic de pneumopathie varricelleuse est porté. Le traitement antiviral, initialement par acyclovir (10 mg/kg/8 h) IV pendant 24 h puis par valacyclovir (1 g 3/j) per os est poursuivi pour une durée totale de 10 jours, accompagné de soins locaux, d'une antibiothérapie par amoxicilline-acide clavulanique(1 g 3 fois/j pendant 7 jours) entreprise devant des signes de surinfection des lésions cutanées. L’évolution clinique a été favorable et les anomalies radiographiques disparaissaient progressivement. Une trachéotomie a été réalisée en vue de faciliter le sevrage de la ventilation mécanique.

## Discussion

La varicelle correspond à la primo-infection par le VZV, appartenant à la famille des Herpes viridae [2, 3]. Il s'agit de la maladie éruptive virale la plus contagieuse, quasi obligatoire de l'enfance, dont les complications sont exceptionnelles chez l'enfant sain [3, 4]. Chez l'adulte, pour des raisons inconnues, la varicelle est plus grave. La (PV) en est la complication la plus fréquente et la plus grave et traduit une dissémination viscérale du VZV [4]. Son incidence est estimée entre 16 et 33% et la mortalité peut atteindre 20 à 50% dans un tableau d’‘dème lésionnel et de défaillance multiviscérale [3–6]. Plusieurs facteurs de risque d'atteinte pulmonaire au cours de la varicelle ont été identifiés: le sexe masculin, l’âge adulte, le tabagisme, une atteinte cutanée comportant plus de 100 éléments, la grossesse, le contact étroit avec un sujet infecté, toute immunodépression [1, 7]. Notre patient présentait plusieurs de ces facteurs de risque (adulte, sexe masculin, tabagisme, atteinte cutanée floride). Rarement symptomatique (moins de 2% des cas), la PV, généralement précédée de quelques jours par une éruption cutanée vésiculo-pustuleuse, peut se manifester par un malaise général avec fièvre, toux, dyspnée, hémoptysie, douleur thoracique de type pleurétique, voire un syndrome de détresse respiratoire aiguë rapidement évolutif et pouvant être mortel [2, 4, 8]. Il n'existe pas de gold-standard pour le diagnostic de pneumopathie varicelleuse; le diagnostic peut être porté devant l'association, sur un terrain à risque, d'une atteinte pulmonaire, dont les caractéristiques radiologiques sont concordantes, et d'une éruption cutanée évocatrice de varicelle, en l'absence d'argument pour une cause annexe Le retentissement gazométrique, notamment l'hypoxémie corrélée à l'importance de la dyspnée, permet d’évaluer le degré de l'atteinte parenchymateuse [4]. L'imagerie thoracique retrouve souvent, des nodules plus ou moins bien limités, diffus, atteignant les deux champs pulmonaires, rarement des opacités hilifuges non systématisées ou des infiltrats hétérogènes [4, 6]. L'endoscopie bronchique peut mettre en évidence des lésions endobronchiques vésiculeuses proximales [6]. Cet aspect particulier est rare, comme en témoigne le nombre limité de cas rapportés dans la littérature. Les mécanismes de formation de cette atteinte ne sont pas encore élucidés. Il pourrait s'agir du même mode de dissémination hématogène que celui impliqué dans les localisations de l'infection à VZV au poumon, au foie, ou au système nerveux central [6]. Les tests microbiologiques (culture virale, méthodes d'amplification génique) réalisés sur les prélèvements du liquide de vésicules ou dans les cellules mononuclées du sang périphérique en période de virémie, ne sont pas nécessaires quand le tableau radio-clinique est typique. Les autres examens (cytodiagnostic, biopsie, sérologie) n'ont pas d'intérêt pratique [9]. Chez notre patient, le diagnostic est retenu devant le contage varicelleux récent, les symptômes respiratoires survenus dix jours après l’éruption cutanée très évocatrice de varicelle, les donnés de l'imagerie thoracique et l’évolution clinique favorable sous antiviraux.

Le traitement curatif recommandé de la PV est à base d'antiviral, par aciclovir de préférence, et par voie intraveineuse (10 mg/kg toutes les huit heures) en cas d'affection sévère, mais aussi par valaciclovir ou ganciclovir pendant une durée de sept à dix jours [1, 6, 8, 10]. Le choix d'une administration par voie intraveineuse, s'il apparaît indiscutable dans les situations sévères de défaillance multiviscérale où l'apport oral est impossible ou l'absorption digestive compromise, doit être mis en balance avec le risque infectieux lié au maintien d'un abord veineux à proximité de lésions cutanées éventuellement surinfectées. Le traitement antiviral diminue la sévérité et la durée de l’éruption cutanée chez l'adulte immunocompétent. Il n'y a pas eu d’étude clinique randomisée permettant de déterminer si le traitement antiviral peut prévenir les complications telles que la pneumonie ou s'il réduit la sévérité de l'atteinte pulmonaire lorsqu'elle est déclarée [6], ainsi que celui de la corticothérapie dans les formes sévères. Ce traitement est souvent accompagné de soins locaux, d'une oxygénothérapie à haut débit et d'une antibiothérapie entreprise devant les signes de surinfection cutanée. L’évolution clinique et radiologique est souvent rapidement favorable du fait de la précocité de la prise en charge. Cependant, des cas de décès dans un tableau d’œdème lésionnel et de défaillance multiviscérale ont été rapportés dans la littérature [3–6].

## Conclusion

La PV est la plus fréquente des complications de la varicelle chez l'adulte. Le diagnostic positif est retenu sur des arguments cliniques, biologiques et radiologiques. Sous traitement antiviral, l’évolution est favorable. L'utilisation précoce et quasi systématique de l'aciclovir en cas de suspicion de PV a entraîné une diminution significative de la mortalité qui ne dépasse guère 1% des cas de PV dans les séries récentes.
